# Magnetic reversal modes in cylindrical nanostructures: from disks to wires

**DOI:** 10.1038/s41598-021-89474-z

**Published:** 2021-05-12

**Authors:** Mariana P. Proenca, Javier Rial, Joao P. Araujo, Celia T. Sousa

**Affiliations:** 1 IFIMUP - Institute of Physics for Advanced Materials, Nanotechnology and Photonics, Physics and Astronomy Department, Faculty of Sciences, University of Porto, Rua do Campo Alegre 687, 4169-007 Porto, Portugal; 2 Instituto de Sistemas Optoelectrónicos y Microtecnología (ISOM), Universidad Politécnica de Madrid, Avda. Complutense 30, 28040 Madrid, Spain

**Keywords:** Nanowires, Magnetic properties and materials

## Abstract

Cylindrical magnetic nanowires are key elements of fast-recording and high-density 3D-storage devices. The accurate tuning of the magnetization processes at the nanoscale is crucial for the development of future nano-devices. Here, we analyzed the magnetization of Ni nanostructures with 15–100 nm in diameter and 12–230 nm in length and compared our results with experimental data for periodic arrays. Our modelling led to a phase diagram of the reversal modes where the presence of a critical diameter (*d* ≈ 30 nm) triggered the type of domain wall (DW) formed (transverse or vortex); while a critical length (*L* ≈ 100 nm) determined the number of DWs nucleated. Moreover, vortex-DWs originated from 3D skyrmion tubes, reported as one of the best configurations for storage devices. By increasing the diameter and aspect-ratio of nanowires with *L* > 100 nm, three reversal modes were observed: simultaneous propagation of two vortex-DWs; propagation of one vortex-DW; or spiral rotation of both DWs through “corkscrew” mechanism. Only for very low aspect-ratios (nanodisks), no skyrmion tubes were observed and reversal occurred by spiral rotation of one vortex-DW. The broad range of nanostructures studied allowed the creation of a complete phase diagram, highly important for future choice of nanoscaled dimensions in the development of novel nano-devices.

## Introduction

The controlled fabrication of magnetic nanowire (NW) arrays with outstanding characteristics is attracting much interest recently owing to their applications in emerging technologies and health cares, largely related to the exhibited anisotropic magnetic properties^1–5^. Among several approaches to prepare NWs, the template-assisted method combining nanoporous anodic aluminum oxide (AAO) with electrodeposition has proved to be a highly efficient and low-cost fabrication procedure^6,7^. However, for data storage or logic circuits applications, it is crucial to understand the factors that determine the reversal mechanism, the switching field, and the thermal stability of these NWs to make them a low-cost alternative to the usual nanostrips produced by lithography^8–12^.

There have been many works studying reversal mechanisms in single particles or arrays of cylindrical NWs^13–17^. Depending on the NWs’ geometry, three main reversal modes have been discussed in the literature: coherent rotation, curling reversal and transverse domain wall (T-DW) propagation. The curling of magnetization in NWs is sometimes called vortex DW-like (V-DW) in the case of strips or pseudo-vortex to avoid the confusion with the strip case^18^. More recently, the designation of Bloch-point wall was proposed for curling in cylindrical NWs^19^. Independently on the adopted designation, the curling mode occurs around the NW axis, allowing a three-dimensional flux-closure where the spins reverse progressively via propagation of a vortex-like (curling) DW. In the particular case when the core and the outer-shell spins are pointing in the opposite direction, one obtains a so-called skyrmion tube in which Block points are formed as hedgehog-antihedgehog pairs^16,20^. The coherent mode refers to homogeneous rotation of magnetization at unison along the total length of the NW; whereas the transverse mode corresponds to nucleation and propagation of a T-DW along the NW’s axis. Other particular designations have also been adopted, such as in-plane and out-of-plane flower like sates^21^, or asymmetric T-DWs^22^.

Analytical calculations have been frequently used to determine the mechanism of nucleation and propagation of DWs in NWs. However, this approach can only qualitatively describe the experimental results^23–26^. On the other hand, micromagnetic simulations have been shown as a powerful technique to study the magnetization reversal modes in individual and arrays of NWs^14,17,27–29^. Since 2001, several works used the micromagnetic simulations to investigate the magnetization reversal process in Ni NWs, with diameters from 10 to 60 nm and lengths from 600 nm to 1 µm^13,30,31^. The authors identified several mechanisms of nucleation and propagation of DWs depending on the Ni NWs geometry and the external applied field. A systematic experimental work performed magnetic measurements simultaneously with micromagnetic simulations identifying T-DW and coherent rotation when the magnetic field is applied parallel and perpendicular to the NW’s axis for Ni NWs with diameter lower than 30 nm. For diameters higher than 40 nm the magnetization reversal changes to a vortex mode in parallel direction^14^. However, these conclusions are not consensual in the literature. For example, Da Col et al.^32^, by combining surface and transmission X-ray magnetic circular dichroism photoemission electron microscopy (XMCD-PEEM) with micromagnetic simulations, reported two well-defined families of DWs: (1) Bloch-point wall type for NWs with more than 90 nm in diameter; and (2) T-DW for smaller diameters. Several other authors used different experimental techniques combined with micromagnetic simulations to reveal the mechanisms of DW propagation, such as electron holography^27,33^, PEEM combined with XMCD spectroscopy^34–36^, magnetic force microscopy^28^, and bright-field transmission electron microscopy and Lorentz microscopy^37,38^. However, several questions are still open, mainly concerning the domain nucleation and propagation in low-aspect-ratio NWs. Only a few reports in the literature were dedicated to the study of NWs with low aspect ratios (AR ≤ 1). Some of them reported for electrodeposited nanostructures in lithographed substrates^21^ and others for preliminary studies in Au/Ni/Au with Ni segment’s lengths from 26 to 640 nm^6,39^.

In this work, we study the switching behavior and reversal mechanisms of a regular hexagonal array of weakly interacting 55-nm diameter cylindrical Ni NWs with lengths between 15 and 100 nm. Magnetic measurements, combined with micromagnetic simulations, provided information on the reversal mechanism of the studied Ni NW arrays. In order to fully characterize the magnetic behavior of such Ni nanostructures, the magnetization reversal process of a wide set of individual cylindrical NWs, with diameters ranging from 15 to 200 nm and lengths from 12 to 400 nm, were also simulated. The results obtained allowed to build a phase diagram of the magnetic reversal modes present in such nanostructures, as a function of their diameter and length. This diagram will provide valuable information for the correct choice of dimensions of magnetic nanodisks/nanowires to be implemented in future devices.

## Results and discussion

**Figure 1:**
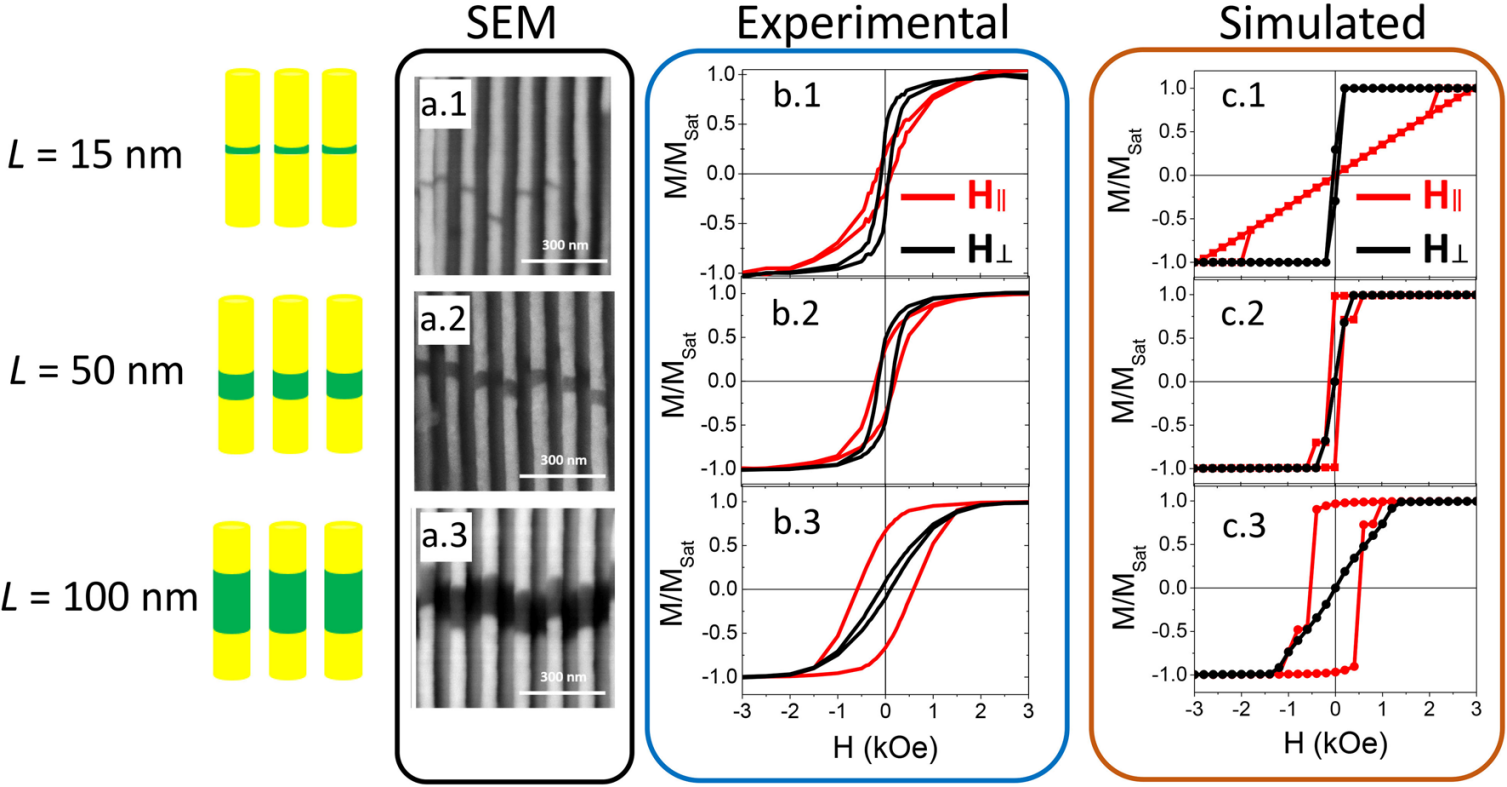
(**a**) SEM cross-sectional images of the nanowires’ length distribution: (**a.1**) *L* = 15 ± 1 nm; (**a.2**) *L* = 50 ± 3 nm and (**a.3**) *L* = 100 ± 4 nm. (**b**) Normalized magnetic hysteresis loops of Au/Ni/Au NWs with lengths (**b.1**) *L* = 15 nm, (**b.2**) *L* = 50 nm and (**b.3**) *L* = 100 nm, with the applied magnetic field parallel (red line) and perpendicular (black line) to the NWs’ long axis. (**c**) Normalized simulated magnetic hysteresis loops of Ni NWs with lengths (**c.1**) *L* = 15 nm, (**c.2**) *L* = 50 nm and (**c.3**) *L* = 100 nm, with the applied magnetic field parallel (red line) and perpendicular (black line) to the NWs’ long axis.

The scanning electron microscopy (SEM) images in Fig. 1a display selected cross-sectional views of the Au/Ni/Au NWs grown in the AAO membrane. The NWs have 55 ± 5 nm in diameter and a center-to-center distance of around 105 nm. The Au non-magnetic segments (brighter sections) have several micrometers in length. The length (*L*) of the magnetic Ni segments (darker sections) was controlled by adjusting the electrodeposition time to obtain several architectures with different aspect-ratios (AR = length/diameter): nanodisks with AR < 1 (*L* = 15 and 25 nm); nanorods with AR ≈ 1 (*L* = 50 nm) and nanowires with AR > 1 (*L* = 100 nm).

The magnetic properties of Au/Ni/Au NWs embedded in the AAO membrane were studied by vibrating sample magnetometer (VSM). Figure 1b shows the magnetic hysteresis loops [*M*(*H*)] recorded for Ni segments with *L* = 15, 50 and 100 nm, with the applied magnetic field parallel and perpendicular to the NWs’ long axis. The sample with 100 nm of Ni in length (Fig. 1b.3) presents a magnetic anisotropic behavior with the easy magnetization axis lying parallel to the wire axis, arising from the competition between the magnetocrystalline anisotropy and the shape anisotropy factors^40^. For the NWs with *L* = 50 nm (Fig. 1b.2) the parallel and perpendicular magnetic loops are very similar, exhibiting low values of coercivity (*H*_c_) and normalized remanence (*m*_r_ = *M*_r_/*M*_Sat_, where *M*_r_ is the remanence and *M*_Sat_ is the saturation magnetization). For Ni segments with *L* = 15 nm (Fig. 1b.1), the *H*_c_ and *m*_r_ decease, presenting similar values in both directions. However even for the smallest aspect ratios (lower than 1) our results evidence the existence of an easy magnetization axis along the parallel direction for all samples, contrary to the reported in the literature^41,42^. This behavior can be attributed to the high dipolar interactions exhibited between the neighbor Ni segments^43^. Even for the case of these samples, with only one Ni segment, the dipolar interactions between the neighboring wires seems to be enough to tilt the easy magnetization axis along the parallel direction.

By comparing the three hysteresis loops shown in Fig. 1b, one can see that *H*_c_ increases with increasing *L* when the magnetic field is applied parallel to the NWs’ axis. However, when the field is applied perpendicular to the NWs’ axis, the *H*_c_ presented the lowest values for *L* = 15 and 100 nm and higher values in the intermediate lengths. The same behavior is observed for *m*_r_ in the perpendicular direction, whereas a monotonous increase of *m*_r_ is shown in parallel direction. The obtained results in perpendicular direction suggest the existence of two different reversal magnetization processes for Ni segments with AR lower and higher that 1.

In order to better understand the role of the dipolar interactions and the mechanism of DW nucleation and propagation, the magnetic hysteresis loops of hexagonal arrays of 7 cylindrical Ni NWs with center-to-center distance of 105 nm were simulated using the Object Oriented Micro-Magnetic Framework (OOMMF) project^44^. The demagnetizing interactions in systems of long NWs are known to be higher than for other arrays of nanostructures^45^, and to increase with decreasing center-to-center distances^46^. To estimate such interacting fields with precision one should simulate arrays with a larger number of wires, which highly increases the simulation time. Hexagonal arrays of 23 nanowires were also simulated but since no major differences were found, all the simulation results presented here were done using arrays of 7 elements in order to reduce the simulation time. The dimensions (length and diameter) of the cylindrical NWs were chosen to replicate the experimental samples studied. Figure 1c shows the simulated magnetic hysteresis loops. As expected, the magnetic properties of NW arrays are mainly dependent on the shape anisotropy of the respective NWs, which is directly related to the aspect ratio (AR) parameter. For AR < 1, both parallel and perpendicular coercivity and remanence are very small, and saturation field is higher when applying the magnetic field along the cylindrical nanowire axis. While for AR > 1, parallel coercivity and remanence are much higher than perpendicular ones, and perpendicular saturation field becomes larger.

Along the perpendicular direction, there is a good agreement between the simulated and experimental results; while along the parallel direction, the agreement is less notorious, especially for AR < 1. In such cases, complex reversal modes may occur, in which the interplay between shape anisotropy and magnetostatic interactions between neighboring wires plays an important role, as will be discussed ahead in more detail. In addition, geometrical factors gain importance at very low aspect ratio structures, such as the curved edges of the electrodeposited nanostructures (instead of flat disks one my encounter curved disks), and the relative location of the disks/cylinders along the z-direction (see SEM images in Fig. 1a).

**Figure 2:**
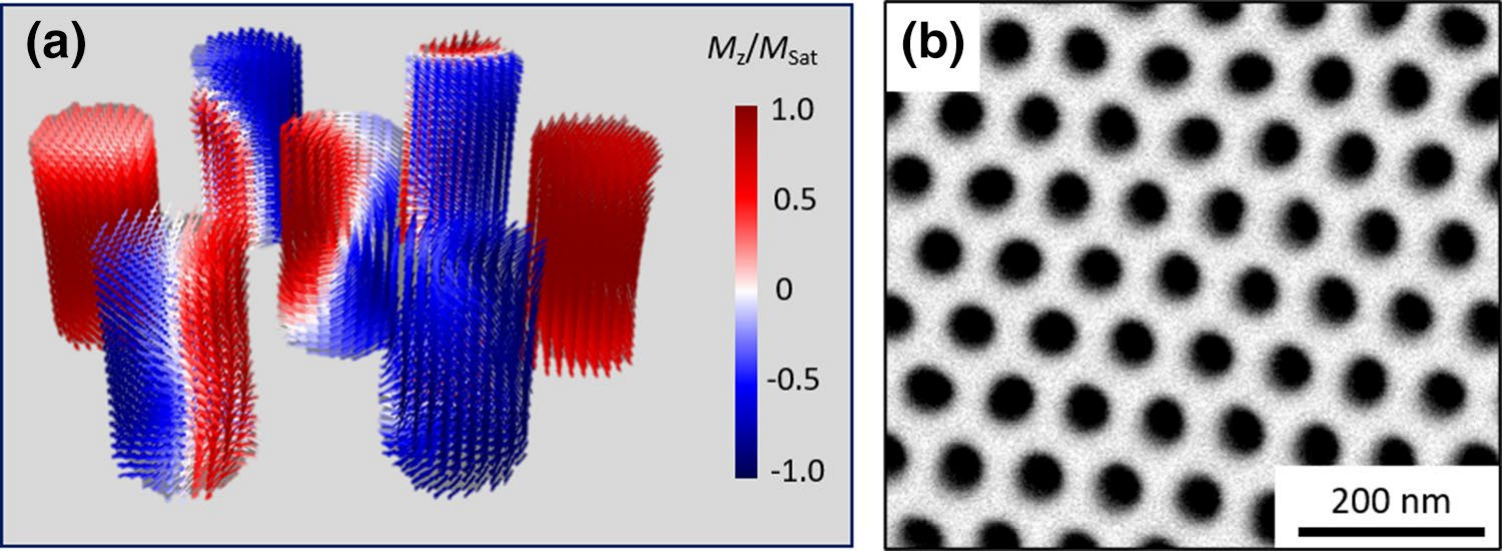
(**a**) 3D representation of the magnetic configuration during reversal of the simulated Ni NW arrays with 100 nm in length illustrating curling rotation with the formation of a vertical vortex when applying the magnetic field along the z-direction (parallel to the NWs’ long axis). (**b**) SEM top view of the template used, evidencing the ordered hexagonal array.

Analyzing the magnetization reversal modes in the simulated arrays of 7 Ni NWs, one can distinguish two main types of reversal: coherent rotation and curling. The reversal mode will depend on the aspect ratio (AR) of the nanostructures. For AR < 1, coherent rotation is observed for both parallel and perpendicular directions of applied magnetic field. For AR ≥ 1, which corresponds to Ni NWs with 50 and 100 nm in length, coherent rotation is again observed along the perpendicular direction; but when applying the magnetic field along the NWs’ length (parallel direction), an unusual vortex rotation is formed. In cylindrical NWs, vortex domain walls (DWs) usually maintain their core axis lying parallel to the NWs’ length. The DWs are formed at the ends of the NW and rapidly propagate towards its center to complete the magnetization reversal^15^. However, in this case, a single vortex is formed during the reversal process (occupying the entire length of the NW) with a vortex core axis lying perpendicular to the NW’s length and changing direction during reversal (see Fig. 2). This type of curling rotation is similar to a so-called asymmetric transverse domain wall^17,22,47^, and will be here referred to as a vertical vortex.

**Figure 3:**
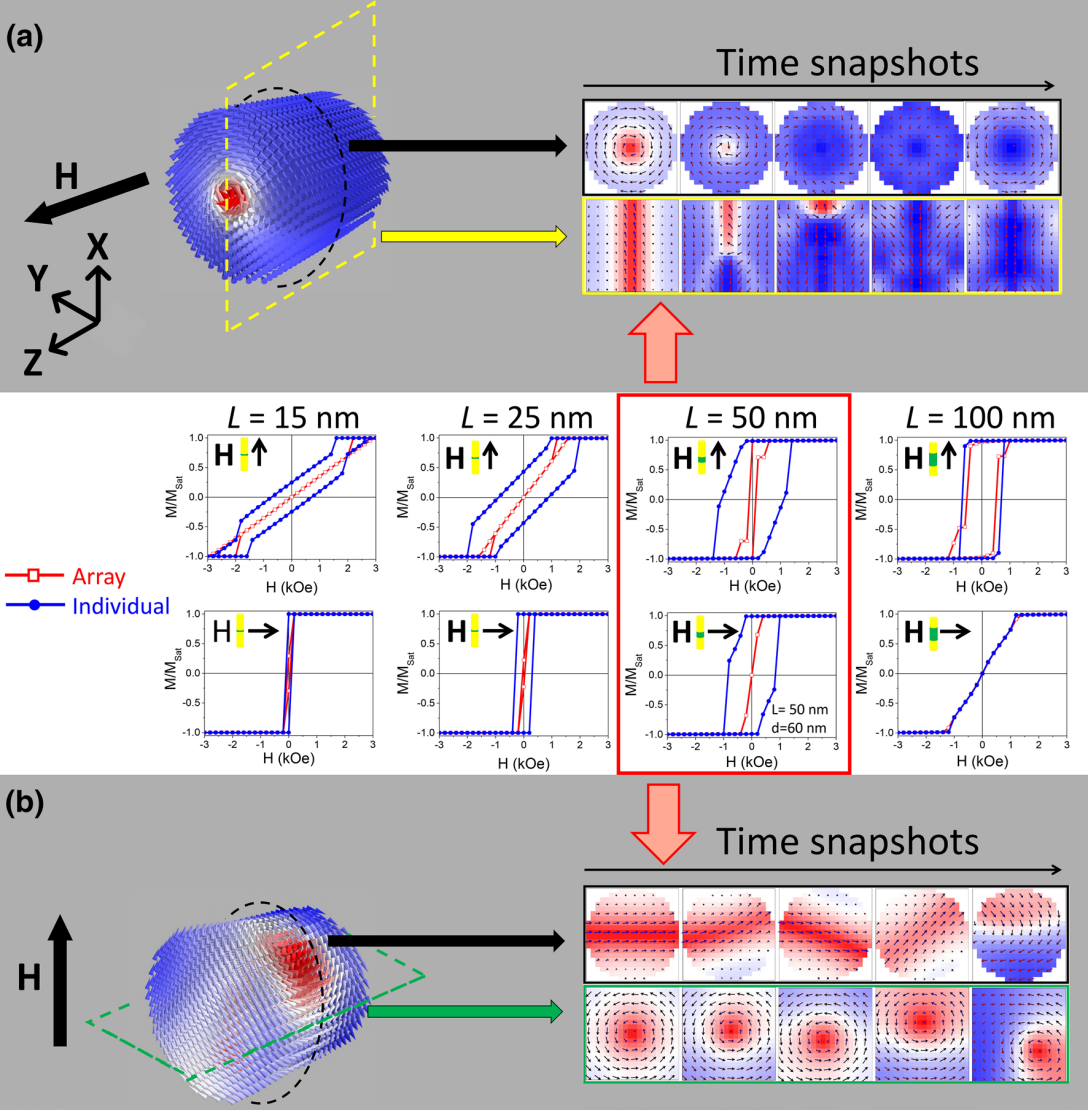
Simulated magnetic hysteresis loops of arrays (red opened symbols) versus individual (blue closed symbols) Ni NWs with 15, 25, 50 and 100 nm in length, when applying the magnetic field parallel (upper plots) and perpendicular (lower plots) to the NWs’ long axis. (**a**) and (**b**) 3D and cross-sectional representations (central x-, y- and z-slices) of time snapshots during magnetization reversal of an individual Ni NW with 50 nm in length and 50 nm in diameter, when applying the magnetic field (**a**) parallel and (**b**) perpendicular to the NWs’ long axis. (**a**) Illustrates the formation of a skyrmion tube and the propagation of a vortex domain wall with alternating chirality from clockwise to counter-clockwise; while (**b**) illustrates reversal by spiral rotation of a vertical vortex. The color scale is the same as in Fig. 2, except that in (**b**) *M*_z_ is replaced by *M*_x_.

To confirm the effect of magnetostatic interactions between neighboring wires in the reversal process, micromagnetic simulations of individual Ni NWs with the same dimensions as the experimentally measured ones were also performed. Comparative magnetic hysteresis loops between the simulated individual NWs and their respective arrays are represented in Fig. 3. All cases show a decrease in remanence and coercivity when NWs are arranged in an array, which can be ascribed to the increased magnetostatic interactions between neighboring elements. Only for *L* = 100 nm (AR > 1) a similar magnetic hysteretic behavior is observed when comparing an individual NW and its respective array, which may be ascribed to the high aspect ratio of the nanostructures. These results show the important role played by both the magnetostatic interactions and shape anisotropy on the magnetization reversal dynamics of cylindrical NW arrays, and thus the impact of correctly tuning the sample’s dimensions, especially for low aspect ratios.

The reversal process also changes when magnetostatic interactions are absent, although it occurs again by two main modes: coherent and curling. When applying the magnetic field perpendicular to the NW’s length, coherent rotation is again observed for all aspect ratios except AR = 1. In this particular case, a vertical vortex is formed with its central core aligned parallel to the applied magnetic field (see Fig. 3b). Reversal then occurs by a complex spiral rotation around the x-axis. When the magnetic field is applied parallel to the cylindrical NW’s axis, all cases displayed a vortex (curling) configuration throughout the entire length of the NW prior to reversal. As the demagnetizing field increases, the outer shell rotates its spins towards the direction of the magnetic field forming a skyrmion tube as illustrated in Fig. 3a^16,17,20,48^. In a skyrmion tube there is a core–shell spin structure pointing on opposite directions. By further increasing the demagnetizing field, a hedgehog skyrmion (Block point or vortex domain wall, V-DW) is formed breaking the inner skyrmion tube core and completing the demagnetization process (see time snapshots in Fig. 3a). Note that during reversal, the vortex configuration changes its chirality from clockwise to counter-clockwise several times (In vortex structures, the relative orientation between its azimuthal component and the core direction defines the chirality of the vortex, while the direction of the core indicates the polarity.).

**Figure 4:**
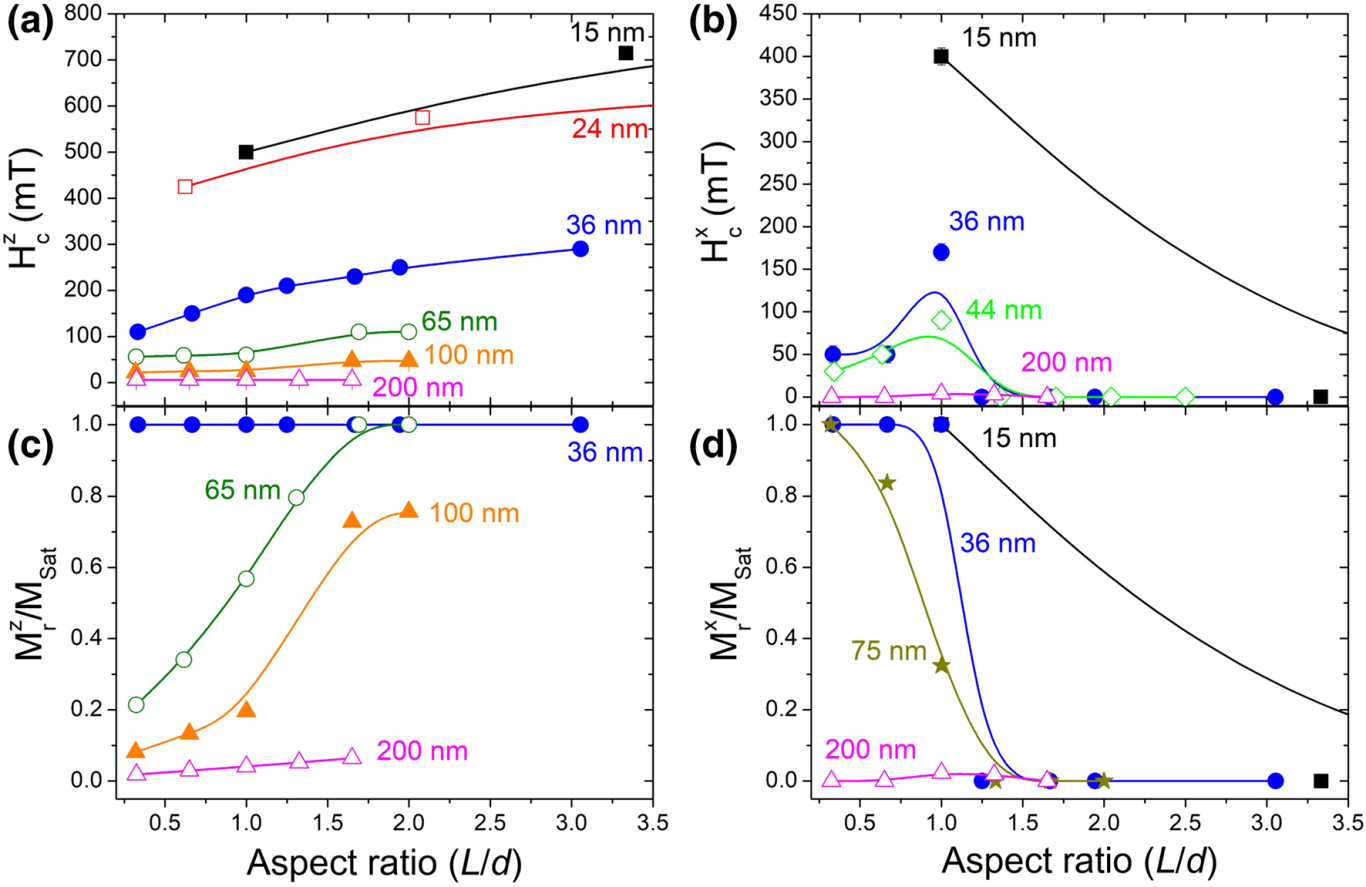
Graphical representations of (**a**,**b**) the coercivity and (**c**,**d**) the reduced remanence dependence on the diameter and aspect ratio of selected simulated Ni individual NWs. The magnetic field was applied (**a**,**c**) parallel and (**b**,**d**) perpendicular to the NW’s length.

The diameter and length of an individual cylindrical NW was seen to highly influence the magnetization reversal process. To fully characterize these reversal modes, micromagnetic simulations of individual Ni NWs with diameters ranging from 15 to 200 nm and lengths from 12 to 400 nm were performed. As expected, parallel coercivity and remanence values increase with decreasing diameters and increasing aspect ratios (see Fig. 4a,c) as hysteresis loops become wider and squared. Similarly, perpendicular coercivity and remanence are also found to increase with decreasing diameters (Fig. 4b,d) as NWs become harder to demagnetize. However, these show a different behavior when varying the NWs’ aspect ratio. Perpendicular remanence decreases with increasing aspect ratio (Fig. 4d), as elongated cylindrical individual NWs have higher demagnetizing fields. While perpendicular coercivity has a maximum at AR = 1 (nanorods or nanodots) for smaller diameters (Fig. 4b), as a vertical vortex is formed, whose high stability increases the applied field needed to reverse the nanostructure’s magnetization (see Fig. 3b).

**Figure 5:**
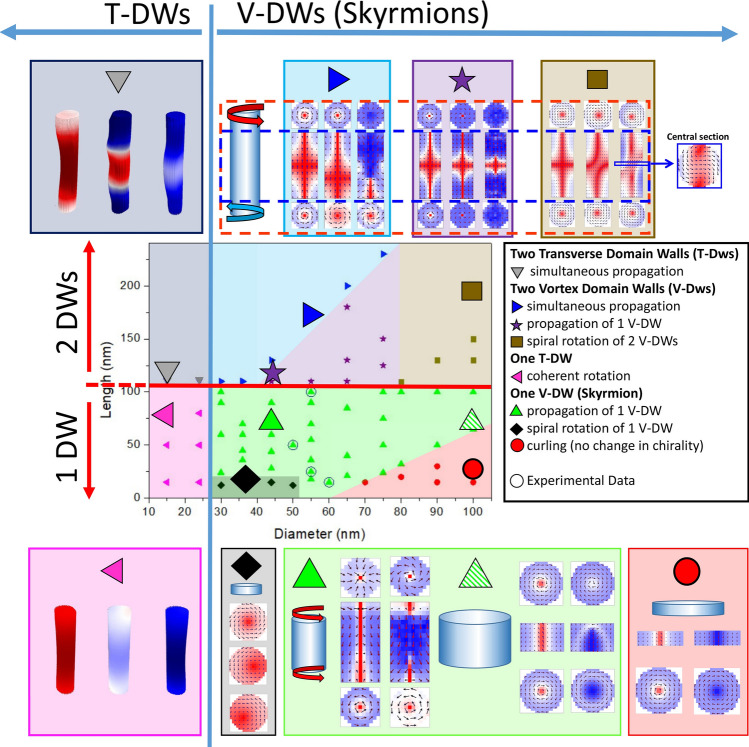
Phase diagram of the magnetization reversal modes in individual Ni nanowires with different lengths and diameters, when applying the magnetic field along the nanowire’s length (parallel direction). Insets show selected cross-sectional representations of the magnetic configuration during reversal (using the same color scale as in Fig. 2).

Finally, a phase diagram of the magnetization reversal modes in individual cylindrical Ni NWs, when applying the magnetic field parallel to the NWs’ length, was created. Figure 5 shows the phase diagram obtained for diameters varying from 15 to 100 nm and lengths from 12 to 230 nm. Colored regions correspond to the different reversal modes identified, while symbols correspond to each simulation performed. The unfilled circles represent the four individual NWs that have the same parameters (length and diameter) as the experimentally measured in this work.

For diameters smaller than a critical value (around 30 nm), magnetization reversal occurs by coherent rotation (pink section of the diagram) or, if the NW is long enough (*L* > 100 nm), by the nucleation and propagation of two transverse domain walls (T-DWs) (grey section of the diagram). In the rest of the cases, curling of the spins occurs prior to reversal due to the cylindrical geometry of the NWs^14^. However, depending on the aspect ratio of the wires (or disks) different modes of curling can be identified.

In general, for lengths higher than 100 nm (and *d* > 30 nm), two vortex DWs (V-DWs) are initially formed with opposite chirality that evolve into two skyrmion tubes with the continuous application of a demagnetizing field (top-right part of the diagram). For small diameters (30 < *d* < 75 nm) and aspect ratios higher than 3, a hedgehog-antihedgehog pair (or Block point) is simultaneously created at each skyrmion core and rapidly move along the wire until complete reversal occurs (shown by blue triangles in Fig. 5)^16,20^. However, if the aspect ratio is smaller than 3, one of the skyrmion tubes increases its length prior to complete reversal, pushing the other skyrmion tube towards the NW's end, annihilating it. Reversal then occurs by the creation of one hedgehog-antihedgehog pair (or Block point) at the remaining skyrmion tube core that rapidly moves along the wire (represented by purple stars in Fig. 5). Recently, it has been shown that the movement of these hedgehogs along the wire produces an emergent electric field, which may be interesting for future technologies^20^. Finally, for large diameters (*d* > 80 nm) and *L* > 100 nm, the two skyrmion tubes with opposite chirality rotate in spirals until complete reversal is achieved (shown by brown squares in Fig. 5). This last reversal process was recently described as a “corckscrew” mechanism in which, for large diameter NWs, a skyrmion tube is formed with a core position in a helical modulation along the wire^49^.

On the other hand, if *L* < 100 nm (and *d* > 30 nm) only one vortex DW is formed, occupying the entire length of the NW and creating a skyrmion tube prior to reversal (green triangles in the diagram)^16,17^. As the demagnetizing field is increased, a hedgehog-antihedgehog pair (Block point) is generated at the skyrmion tube core that then moves towards the NW's ends. After the complete propagation of the hedgehogs, a curling rotation (single V-DW state) is present along the wire. If *d* > 60 nm and AR ≲ 0.3, complete reversal occurs by curling rotation with no change in chirality (shown by red circles in Fig. 5). Otherwise, the chirality of the V-DW is seen to change from clockwise to counter-clockwise several times until complete reversal is achieved (see Fig. 3a).

The only exception occurs for the particular case of nanodisks with 30 < *d* < 50 nm and *L* < 15 nm, where no skyrmions have been observed (represented by black diamonds in Fig. 5). Instead, when increasing the demagnetizing field, the single V-DW core starts rotating in a spiral with increasing diameter (keeping itself always parallel to the NW axis), until reversal occurs by a coherent spiral rotation. This rotation mode is similar to the asymmetric transverse DW found for NW diameters close to the transition value between the vortex and the transverse DWs^17^.

## Conclusions

Au/Ni/Au nanowire arrays, with around 55 nm in diameter, having Ni length from 15 nm up to 100 nm were synthesized by template assisted electrochemical deposition into the pores of AAO templates. Magnetic hysteresis loops, measured at room temperature, showed a clear magnetic anisotropic behavior for high aspect ratio wires (AR > 1), with large coercivity and remanence values when applying the magnetic field parallel to the wire axis. While for AR < 1 the parallel and perpendicular magnetic loops were very similar, exhibiting low values of coercivity and remanence. Either way, our results evidenced the existence of an easy magnetization axis along the parallel direction for all samples, which can be attributed to the high dipolar interactions exhibited between neighboring elements.

Micromagnetic simulations were performed for both Ni nanowire arrays and individual wires, replicating the magnetic hysteresis loops obtained. These also allowed a better understanding of the magnetization processes occurring during reversal and evidenced the important role played by both the magnetostatic interactions and shape anisotropy on the magnetization reversal dynamics of cylindrical NW arrays. In particular, a vertical vortex was formed during reversal of an array of 7 Ni NWs with around 50 nm in diameter and 100 nm in length, while a skyrmion tube was observed if the same NWs were isolated.

Finally, a phase diagram of the magnetization dynamics during reversal of individual cylindrical Ni NWs was created using micromagnetic simulations. In general, bellow a critical diameter (around 30 nm for Ni), coherent rotation is observed, while above such critical diameter, curling of the spin structure occurs. Then, if *L* > 100 nm, two domain walls (DWs) are formed, either transverse DWs (below the critical diameter) or vortex DWs (above such critical diameter). The curling reversal dynamics (or vortex DW propagation) was also found to highly depend on the nanostructures’ dimensions. For *L* > 100 nm two skyrmion tubes with different chiralities were formed, which propagated in different ways depending on the diameter of the wires. While for *L* < 100 nm only one skyrmion tube was formed, which changed chirality during reversal for small diameters and kept its chirality unaltered for larger diameters. The only exception was found for very low aspect ratio wires (nanodisks) where a single V-DW was formed and reversal occurred by a coherent spiral rotation of the V-DW core.

In summary, the magnetic properties of cylindrical nanostructures were found to highly depend on their geometrical ratios. As most of the applications of cylindrical magnetic nanowires need a correct understanding of their magnetic reversal processes, it becomes highly important to accurately understand and fully characterize in advance the magnetic behavior of these nanostructures. This work will thus highly contribute to the correct choice of dimensions of Ni nanostructures aiming their implementation in future devices.

## Materials and methods

AAO templates were prepared by electrochemical oxidation of high-purity (> 99.997%) Al foils. A two-step anodization procedure was used in order to achieve the high organization and pore structure homogeneity^7^. The first anodization was performed in oxalic acid 0.3 M at 40 V and 4 °C during 24 h. The resulting porous-oxide layer was then etched with 0.5 M phosphoric/0.2 M chromic acids at 40 °C for 12 h. A second anodization was performed with the same conditions as the first one during 20 h resulting in an AAO template thickness of around 50 µm. The remaining Al foil was then removed by chemical etching in an aqueous solution of 0.2 M CuCl_2_ and 4.1 M HCl at room temperature. Finally, the alumina barrier layer at the bottom of the pores was removed using 0.5 M H_3_PO_4_, thus obtaining AAO templates with opened pores of around 50–60 nm in diameter.

Potentiostatic electrodeposition was used to grow Au/Ni/Au multisegmented NWs inside the AAO pores. First, a thin Au layer was sputtered at the bottom of the template to serve as the working electrode in a three-electrode cell. A Pt mesh served as the counter electrode, and Ag/AgCl (in 4 M KCl) as the reference electrode. The Au segment was deposited at − 1.7 V at room temperature with a deposition rate of 2.5 nm/s using Orosene E 4 g/L (from Italgalvano SPA). Then the Ni layer was deposited at − 1.5 V using a mixture of NiSO_4_·6H_2_O (350 g/L), NiCl_2_·6H_2_O (45 g/L) and H_3_BO_3_ (45 g/L) with a deposition rate of 15 nm/s at 40 °C. After the Ni deposition, the second Au segment was deposited using the same conditions as the first one. The noble metal layers were used to avoid any Ni oxidation and to promote homogeneous nucleation of the Ni segment.

The morphology of the produced NWs was analyzed by scanning electron microscopy (SEM, FEI Quanta 400FEG Field Emission). Ni segment’s dimensions and size distributions were evaluated with *ImageJ* open software^50^. The magnetic behavior of the multisegmented Au/Ni/Au NW arrays embedded in the AAO template was studied by means of a commercial Oxford Instruments 1.2 T resistive vibrating sample magnetometer (VSM) at room temperature with applied magnetic field [*M*(*H*)*;* from 10 to − 10 kOe]. The magnetic hysteresis loops [*M*(*H*)] were measured with the field (*H*) applied along the parallel (*H*^‖^) and perpendicular ($H^{ \bot }$) directions with respect to the NWs’ longitudinal axis.

The magnetic reversal mechanism of individual cylindrical Ni NWs and respective hexagonal arrays of 7 NWs, were analyzed by micromagnetic simulations using the Object Oriented Micro-Magnetic Framework (OOMMF) project^44^. The Ni saturation magnetization and stiffness constant values used were 490 × 10^3^ A/m and 9 × 10^–12^ J/m, respectively. All simulations considered a stopping condition of |d*m*/d*t*|= 1 deg/ns and a damping factor of 0.015. A parallelepipedic mesh was used with unit cell sizes tuned between 1 and 5 nm, depending on the respective length (*L*) and diameter (*d*) simulated. For the magnetic hexagonal arrays, an interwire (center-to-center) distance of 105 nm was used and the unit cell sizes were all tuned to 5 nm (to reduce the simulation time).

## Data Availability

The datasets generated and analysed during the current study are available from the corresponding author upon reasonable request.
